# Behavioural Plasticity by Eastern Grey Kangaroos in Response to Human Behaviour

**DOI:** 10.3390/ani9050244

**Published:** 2019-05-15

**Authors:** Caitlin M. Austin, Daniel Ramp

**Affiliations:** Centre for Compassionate Conservation, Faculty of Science, University of Technology Sydney, Ultimo, NSW 2007, Australia; Daniel.Ramp@uts.edu.au

**Keywords:** adaptation, behavioural plasticity, eastern grey kangaroos, grouping behaviour, human behaviour, human shield, hunting

## Abstract

**Simple Summary:**

Many species of wildlife live in landscapes they share with people. Some exploit resources and protection provided by close proximity to people, while others learn to avoid people all together. In this study, we sought to test whether individuals from a population of eastern grey kangaroos altered grouping and spacing behaviour in response to human presence, depending upon whether the intent and actions of those people were benign or harmful. Under harmful conditions, kangaroos failed to form larger groups when far from cover, however, this typical antipredator grouping behaviour persisted when human disturbances were benign. These differences in grouping and spacing behaviour suggest that kangaroos can exhibit bidirectional behavioural plasticity at fine scales, a trait that may confer adaptive advantages when sharing landscapes with humans.

**Abstract:**

Sharing landscapes with humans is an increasingly fraught challenge for wildlife across the globe. While some species benefit from humans by exploiting novel opportunities (e.g., provision of resources or removal of competitors or predators), many wildlife experience harmful effects, either directly through persecution or indirectly through loss of habitat. Consequently, some species have been shown to be attracted to human presence while others avoid us. For any given population of a single species, though, the question of whether they can recognise and change their response to human presence depending on the type of human actions (i.e., either positive or negative) has received little attention to date. In this study, we chose to examine the behavioural plasticity within a single population of eastern grey kangaroos (*Macropus giganteus*) to both positive and negative human activity. Within a relatively small and contiguous landscape, we identified areas where kangaroos experience a combination of either low and high frequencies of benign and harmful human disturbances. From six sampling sessions over five months, we found that density and group sizes were higher where humans acted benignly towards them, and that these groups had higher representations of sub-adults and juveniles than where humans had harmful intentions. Importantly, we found that the vital antipredator strategy of increasing group size with distance from cover was not detectable at sites with low and high levels of harm. Our findings suggest that these kangaroos are recognising and adjusting their behavioural response to humans at fine spatial scales, a plasticity trait that may be key to the survival of these species in human dominated landscapes.

## 1. Introduction

The global decline of mammals has been driven by a combination of increasing modification and urbanisation of landscapes [1] and the exploitation and forcible exclusion of free-roaming animals [2]. This is particularly apparent for large mammals, many of which have declined, are considered threatened, or have gone extinct [3,4]. Of the remaining species, some are maintaining populations despite pressure from habitat loss, increasing fragmentation, and climate change, albeit at lower densities than in recent history [5]. However, the persistence of populations at regional scales masks the complexity of challenges these species face when adjusting to local processes. The ability to make the most of novel opportunities at local scales may be as valuable as the ability to avoid or survive threats. Cognitive learning through individual and collective experiences of extrinsic processes is a key survival mechanism, facilitating both the acquisition of temporally and spatially variable resources and the ability to reduce uncertainty in risk assessment of threat signals. Similarly, differences in risk taking can also be shaped by personality traits held by individuals, conditioned by innate temperaments [6]. There is growing evidence that persecuted species of large mammals show adaptive responses to hunting [7,8,9,10,11], driven by learning and selection processes [12,13]. While animals have been shown to distinguish and adjust behaviourally to different levels of threats posed by hunters [11,14], evidence for plasticity in response to both positive and negative behaviour by humans, which may be key to long term persistence, has so far received little focus.

Many species exhibit fear responses towards humans, often eliciting antipredator responses greater than that of their natural predators [15,16,17]. With a rapidly expanding human population, community dynamics have shifted to accommodate humans as ‘super predators’ [18]. This effect has been particularly clear for hunted species like deer (*Cervus elaphus*), giraffes (*Giraffa camelopardalis tippelskirchi*), and wild boars (*Sus scrofa*), where hunting alters sex ratios [19,20], demography [21,22], habitat use [20,23], and behavioural patterns [24]. However, humans can also provide benefits for many species, offering protection [7,8,9] and foraging opportunities [10]. This effect can be direct, whereby species are attracted to urban zones to exploit novel resources and habitats [25], or else indirect by exploiting fear in others (e.g., the use of humans as a shield against predators) [7,9]. Antelope (*Tragelaphus buxtoni*) have been shown to relocate to nearby human settlements to exploit lower densities of spotted hyenas (*Crocuta crocuta*) [9], while pregnant moose (*Alces alces*) select birthing sites nearer to roads to shield mothers and young from brown bears (*Ursus arctos*) [7]. These situations are not always binary: responding to one threatening process can expose populations/species to other stressors [25]. For example, roe deer (*Capreolus capreolus*) seek human settlements as a shield against predators but must trade-off the increased risk of poaching encountered in urban zones [23,26,27]. These examples show that animals are able to differentiate between different levels of threat and modify their behaviour accordingly. Furthermore, recent evidence suggests that some species can adjust their response to the presence of the same predator in opposing directions. Coyotes (*Canis latrans*) have been shown to alter their response to human disturbance, limiting exploratory behaviour in rural landscapes where they are regularly persecuted, while becoming bolder in urban settings where humans pose little threat and provide anthropogenic foods [28].

In Australia, eastern grey kangaroos (*Macropus giganteus*) are a large free-ranging mammal hunted by humans that also experience high levels of human presence with benign intent, making this species ideal for modelling how free-living mammals respond to contrasting levels of human interaction. Eastern grey kangaroos are a gregarious woodland species [29,30,31] that form open-membership fission-fusion groups [32,33]. Group composition changes as they move through the landscape, forming larger groups in the morning and afternoon while foraging in open areas and breaking down into smaller groups during rest times in the middle of the day [34]. Increasing group size in eastern grey kangaroos has been shown to be an antipredator response, implemented when foraging in open areas to reduce the risk of predation [35,36,37]. Forming larger groups enables prey species to detect threats sooner through the many-eyes hypothesis [38,39] and benefit from the dilution effect where the probability of attack decreased as group size increases [32,35,40,41]. Eastern grey kangaroos are prey for foxes (primarily juveniles) [35] and dingoes [42], which are capable of limiting population growth [42,43]. It has been reported that group sizes vary with the availability of resources [44], distance from safety [36,37], and predation risk [35,36], but there is little knowledge of the effect of human disturbance on group size.

Eastern grey kangaroos are legally hunted throughout the majority of their range in eastern Australia, either for commercial harvest [45] or under licenses for damage mitigation [46]. However, illegal hunting is common, with shooting taking place on private properties either by landholders or trespassers hunting for sport. Interactions between humans and kangaroos are not always negative, as kangaroos can find safety and resources at highly frequented tourist locations, such as camp sites and picnic areas in national parks, as well as reserves, golf courses, and sporting ovals [47]. Kangaroos may be exploiting these locations as the high frequency of humans showing kindness or benign interest typically excludes human hunters and natural predators [48]. The behaviour of humans towards kangaroos has not been comprehensively studied and there is no study that has examined whether kangaroos modify their behaviour in response to human hunting or benign disturbances.

We suggest that differences in response to human presence may be being driven by both the frequency of interactions (high or low) and the intent of those interactions (positive or negative). The aim of this study was to collect empirical evidence of behavioural plasticity of kangaroos to human presence when varying in both of these aspects, frequency and intent. In particular, the study was designed to test whether these patterns suggest kangaroos are able to adjust responses at fine scales, which would infer learning capacity. The study was not designed to explicitly differentiate between learning and selection (sometimes referred to as sorting), nor was it manipulative in an effort to identify plasticity among specific individuals. Rather, our goal was to quantify bi-directional (fear and attraction) behavioural plasticity in responses to human presence in a large mammal at fine scales. To achieve this, we recorded grouping and spacing behaviour in a population of eastern grey kangaroos that experiences different combinations of low and high frequencies of positive (or benign) and harmful human disturbances.

## 2. Materials and Methods

### 2.1. Study Area

**Figure 1:**
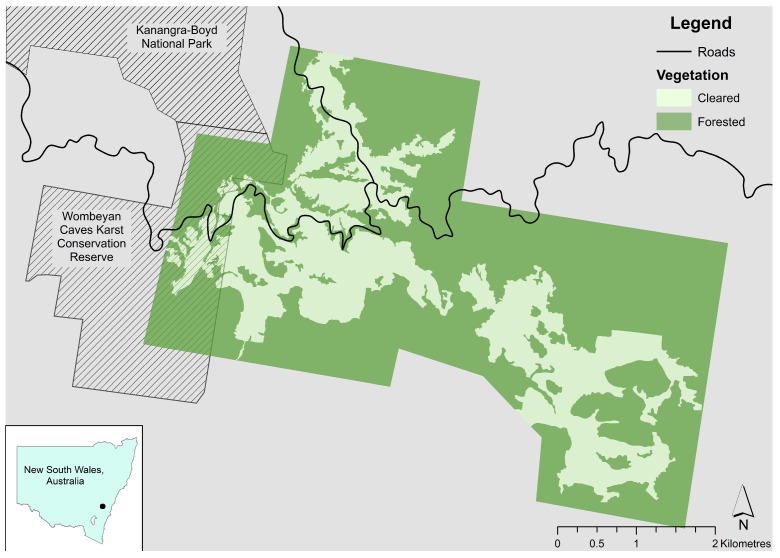
Location of study area within New South Wales, Australia, showing roads and forested and cleared areas within the study area. Property boundaries and human disturbance were omitted to ensure anonymity.

We located a free-ranging population of eastern grey kangaroos residing in the surrounds of Wombeyan Karst Conservation Reserve in the Southern Highlands of New South Wales (NSW), adjacent to Kanangra-Boyd National Park (Figure 1). The reserve and national park are surrounded by private properties with a mix of cleared land and forest. Once we located the general region of study, we informally interviewed landholders and national park staff on the patterns of human movement and whether they were wildlife friendly or allowed hunting. We use the term hunting here to refer to any legal or illegal activities that result in kangaroos being shot, to ensure direct comparison with similar studies elsewhere where hunting is also done as a sport. Contiguous private properties and the reserve were chosen because they were similar in habitat and were frequently used by eastern grey kangaroos which could move freely across the entire area. The total area of the study area encompassed approximately 850 hectares, presenting a unique opportunity to quantify responses of kangaroos to high or low frequencies of human interactions that were either well-intentioned and benign, or else harmful.

Quantitative evidence of the manner of disturbance (either positive or negative) was not obtainable due to safety concerns in areas of frequent hunting (hunters were not receptive to participating in data collection). However, we obtained permission to deploy motion-sensing camera traps for two months to confirm the disturbance activities taking place on each property, allowing us to describe properties as either high (greater than one interaction per week) or low frequency of human interaction (less than one interaction per week). The reserve was chosen as tourists and park staff frequently interacted with kangaroos at the Wombeyan Caves campground, an open expanse of cleared land covering 17.1 hectares, where human activity either ignored the kangaroos (benign) or else was well intentioned (e.g., photography). We classified this location as High Benign. Some privately managed areas within the study area were wildlife friendly, a total of 232.4 hectares of cleared land. These areas discouraged trespassers, especially hunters, kangaroos were left alone, and the frequency of interactions were low. We considered these locations as Low Benign. In contrast, there were privately managed areas where kangaroos experienced harmful disturbances, either through hunting or chasing, where the intent was to cause harm. The frequency of these interactions was either less than weekly, Low Harm (104 hectares), or greater than once per week (High Harm). High Harm areas typically saw regular shooting and covered 139 hectares of cleared land surrounded by forest.

### 2.2. Kangaroo Surveys

On-foot surveys were conducted between October 2016 and February 2017. Cleared areas across the entire area were surveyed six times on fair-weather days with low wind. As eastern grey kangaroos are crepuscular [33], surveys were conducted either between 0600–0830 or 1630–1900, when kangaroos were most likely to be grazing in the open. Surveys consisted of systematically and covertly traversing all cleared areas on foot, hugging the tree line to avoid detection. Upon sighting an eastern grey kangaroo, video and photographs were recorded using a digital camera (Canon EOS 70D Digital SLR with Canon EF100-400 mm lens). Spatial coordinates of the observation location were recorded using a GPS (±5 m) and the bearing and distance to the individual were recorded using a Bushnell rangefinder (±0.9 m). Spatial coordinates for each individual were derived from these measurements and were imported into ArcGIS (v10, 2016 Esri). Surveying was conducted in a manner to ensure individuals were not record twice in a session, however, individuals were not identifiable between sessions.

Grazing density was defined as the total number of kangaroos surveyed within a given session per square kilometre of cleared habitat. Group membership is typically ascertained by applying nearest neighbour distance rules, with a variety of distances applied under different circumstances, herein described as the ‘chain-rule’. Using ArcGIS, individuals were assigned to a group using three different distances frequently reported in the literature for eastern grey kangaroos; 15 m [32,41,49,50], 30 m [35,37,51,52], and 50 m [34]. Pouch young were not included in the total count of group size [34] unless they were out of the pouch.

### 2.3. Group Size, Clustering and Demography

Eastern grey kangaroos are known to exhibit antipredator responses that result in strong correlations between group size and distance to cover [35,36,37]. If humans are viewed as threats, we predicted that group size should increase with increasing distance from cover. Increasing group size with threat level (i.e., further from cover), would be expected under the ‘many-eyes hypothesis’ and conforms to landscape of fear theory. We predicted that kangaroos would avoid areas with frequent harmful interactions with humans, resulting in lower densities than those experiencing less disturbance. We would also expect to see groups more tightly clustered where human threat is higher. Conversely, attraction to the safety that positive human intentions create by shielding individuals from hunters or other predators conforms to the ‘human shield hypothesis’ [7]. If humans can also be viewed as providing a shield from other predators (including hunters), then we predicted that there would be higher densities and larger group sizes of kangaroos where human presence is higher (attraction), with the distance from cover relationship continuing to hold and looser group clustering.

To obtain a quantitative measure of clustering within each group we calculated the nearest neighbour distance for all individuals from groups with a group size >1. Geodesic distances between each individual’s nearest neighbour were measured in ArcGIS (v10.4, 2016 Esri) using the ‘near table’ tool. We used the average nearest neighbour distance for each group as a metric of group clusteredness. These measurements were conducted on groups determined using all three measures of chain-rule; 15 m, 30 m and 50 m.

**Figure 2:**
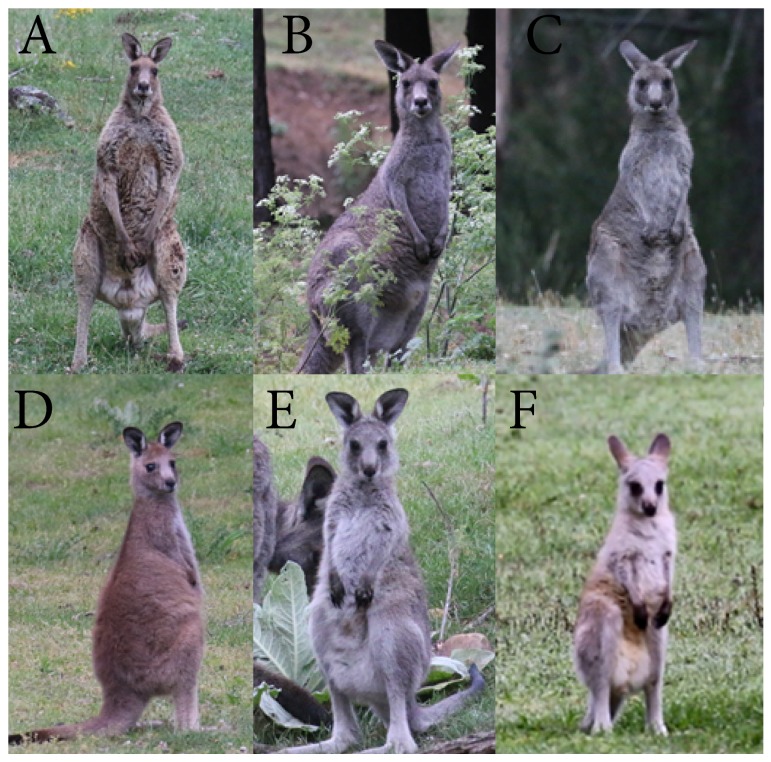
Demography classification reference images of (**A**) large adult, (**B**) medium adult, (**C**) small adult, (**D**) sub-adult, (**E**) young-at-foot, and (**F**) pouch young.

To test for demographic differences across disturbance types, individuals were assigned to size/maturity categories using photographs; large adult, medium adult, small adult, sub-adult, young-at-foot, and pouch young (Figure 2). A random subset of 100 photographs were validated by an independent assessor familiar with eastern grey kangaroos; consensus was reached for all 100 individuals. The demographic composition of each group was calculated as a proportion of the total group size including joeys in the pouch. The proportions of each demographic category were averaged across groups and sampling sessions for each disturbance type at three definitions of chain-rule (15 m, 30 m, and 50 m).

Using the 15 m chain-rule we determined the position of mothers, young-at-foot, and pouch young (vulnerable individuals) within the group with respect to forested cover. Position was classified as either in front or behind and was determined by measuring the distance between both the individual (IDC) and the group centre (GDC) from forested cover. The geometric centre for each group was calculated in statistical package ‘rgeos’ [53], R v3.5.1 (R Core Team, 2018). Distances were measured in ArcGIS using the ‘near table’ tool and applying the geodesic method parameter. Subtracting IDC from GDC yielded a positive or a negative value, where positive values reflected vulnerable individuals positioning themselves closer to the forest edge than the group centre, and negative values further away.

### 2.4. Landscape Characteristics

Eastern grey kangaroos use forested habitat as a refuge and forage closer to cover when predation risk is high [35,36,37]. The position of a group from forested cover was calculated in ArcGIS from the geometric centre of the group for all chain-rules. Foraging and patch choice by eastern grey kangaroos is strongly associated with resource quality [54,55,56]. Kangaroos typically prefer green grass [44,57,58] owing to its higher energetic value [59]. Grass quality at the centre of each group of kangaroos was quantified by determining the relative green channel brightness (greenness) of vegetation from digital photographs. Due to the high correlation between greenness and biomass [60], resource quality was inferred by the greenness of resources for each group of kangaroos. Following Richardson et al. [61], colour channel information (digital number) for red, green, and blue channels were extracted for each pixel in the region of interest using the ‘raster’ package [62] in the program R [63]. Total brightness was calculated as the sum of the three colour channels for all pixels which was in turn used to calculate the relative green channel brightness (greenness).

### 2.5. Statistical Analysis

We conducted a one-way analysis of variance to test for differences in grazing density between disturbance types, averaged across sampling sessions. Data were log transformed to satisfy assumptions of normality and homoscedasticity. We conducted TukeyHSD to examine the differences between the four disturbance types; High Benign, Low Benign, Low Harm, and High Harm. To detect distributional skew or kurtosis in group size data from each disturbance type and chain-rule we ran D’Agostino test of skewness and the Anscombe–Glynn test of kurtosis from statistical package ‘moments’ [64]. Differences in mean group size determined by the three chain-rules were examined using linear mixed models from the statistical package ‘lmer4’ [65]. To test our hypotheses, we analysed the effect of disturbance type and chain-rule on logged group size, with sampling session as a random variable. To test for differences in group size across disturbance types within and between chain-rules we ran pairwise least-square means comparisons using the ‘lsmeans’ package [66]. Similarly, we used linear mixed models to test for differences in clustering across disturbance types using likelihood ratio tests and multiple comparisons of means with Tukey contrasts from statistical package ‘multcomp’ [67]. The clustering metric (mean nearest neighbour distance) was log transformed prior to analysis to satisfy assumptions of normality. Separate models were run for each chain-rule with sampling session and group size as random variables. To test for demographic differences across disturbance types we ran a series of linear mixed models for each demographic category with session and group size as random variables. These analyses were applied separately to data resulting from different measures of chain-rule; 15 m, 30 m, and 50 m. Inference was conducted with likelihood ratio tests and multiple comparisons of means with Tukey contrasts. To determine whether the positioning of vulnerable individuals varied among disturbance types, the proportion of individuals occurring either closer or further from forest edges was calculated for groups, classified into 20 m brackets of distance from cover. To determine if there was a significant difference between disturbance types we ran generalised additive models with disturbance as a fixed factor and distance to cover as a smoothing factor. A series of models were run with each disturbance type as the reference level (intercept).

Generalised linear mixed models were run to test predictions of the response of group size to distance from cover. Logged group sizes were regressed against logged distance from cover, nested within disturbance types, with sampling session as a random variable. Using a negative binomial function, we ran these models for three different chain-rules. Statistical inference was conducted by assessing 95% confidence intervals which were estimated using Laplace approximation [68]. Similarly, to test for differences in resource greenness across disturbance types we ran linear mixed models with disturbance type as a fixed factor and sampling session as a random variable. Inference was conducted using likelihood ratio test and multiple comparisons of means to determine which disturbance types were statistically different from one another. Linear mixed models were also used to test the response variable of logged group size to resource greenness, nested within disturbance type and with sampling session as a random variable. Confidence intervals were estimated using Laplace approximation. All analyses were conducted in R v3.5.1 [63].

## 3. Results

### 3.1. Grazing Densities and Group Sizes

**Figure 3:**
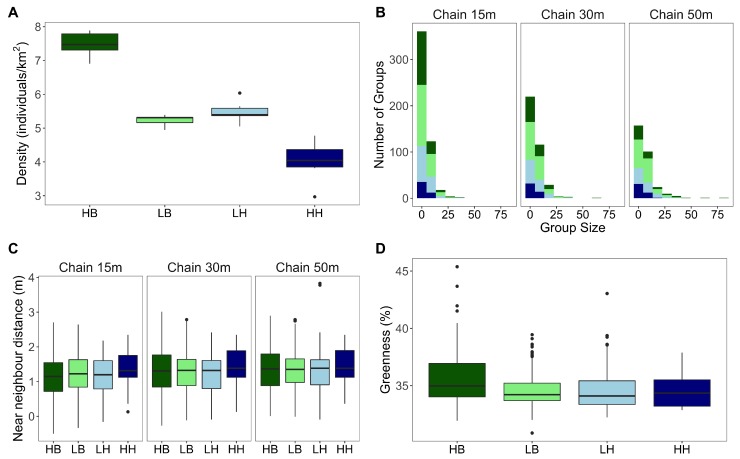
(**A**) Logged grazing density of eastern grey kangaroos in cleared habitat across different types of human disturbance. (**B**) Logged group size as a function of disturbance and different chain-rule (15 m, 30 m, and 50 m). (**C**) Logged nearest neighbour distance per group across disturbance types of human disturbance and at each chain-rule; 15 m, 30 m, and 50 m. (**D**) Resource greenness across disturbance types, error bars indicated standard error. Groups were determined using the 15 m chain-rule. For all plots human disturbance was expressed as an abbreviation; HB: High Benign (dark green), LB: Low Benign (light green), LH: Low Harm (light blue), HH: High Harm (dark blue).

A total of 2228 kangaroos were recorded across the six sampling sessions; the mean number of individuals recorded each session was 368.2 (±14.2). Nineteen kangaroos were disturbed during data collection; these individuals were included in density analysis but were removed from all other analyses. Disturbance type had a significant effect on grazing density (F${}_{3,20}$ = 74.83, *p* < 0.001) (Figure 3A). On average, there were 2 kangaroos more per square kilometre at High Benign (*HB*) sites than at Low Benign (*LB*) (*p* < 0.001) and Low Harm (*LH*) sites (*p* < 0.001), while there were around 3.5 fewer individuals per square kilometre at High Harm (*HH*) sites (*p* < 0.001). There was no significant difference in grazing density between *LH* and *LB* sites (*p* = 0.714). This trend was mirrored by group sizes, where more groups were consistently observed at both benign sites relative to harm sites (Figure 3B). Group size data for all disturbance types exhibited a positive skew (>1) (Table 1), which was stronger for benign disturbances as the presence of large groups sizes (>25 individuals) resulted in longer right tailed distributions (Figure 3B). Distributions for all disturbances were leptokurtic, exhibiting a strong degree of “peakedness” resulting in high positive kurtosis estimates (>3) (Table 1). Both skewness and kurtosis decreased with increasing chain-rule as smaller groups became consolidated (Table 1), shifting the distribution to the right and reducing the peak (Figure 3B). However, this trend was not consistent for *HH* sites, where the merging of smaller groups using the 50 m chain-rule resulted in a stronger skew and kurtosis than observed when the 15 m or 30 m chain-rules were used (Table 1). Despite this, there was no significant difference in mean group sizes across disturbance types, except using 50 m chain-rule which resulted in significantly larger group sizes at *HB* than *HH* (*p* = 0.003).

### 3.2. Clustering and Demography

We did not detect an effect of disturbance type or chain-rule on the average distance between individuals within a group (15 m: *p* = 0.158, 30 m: *p* = 0.560, 50 m: *p* = 0.853) (Figure 3C), although mean near neighbour distances were highest at high harm sites.

**Figure 4:**
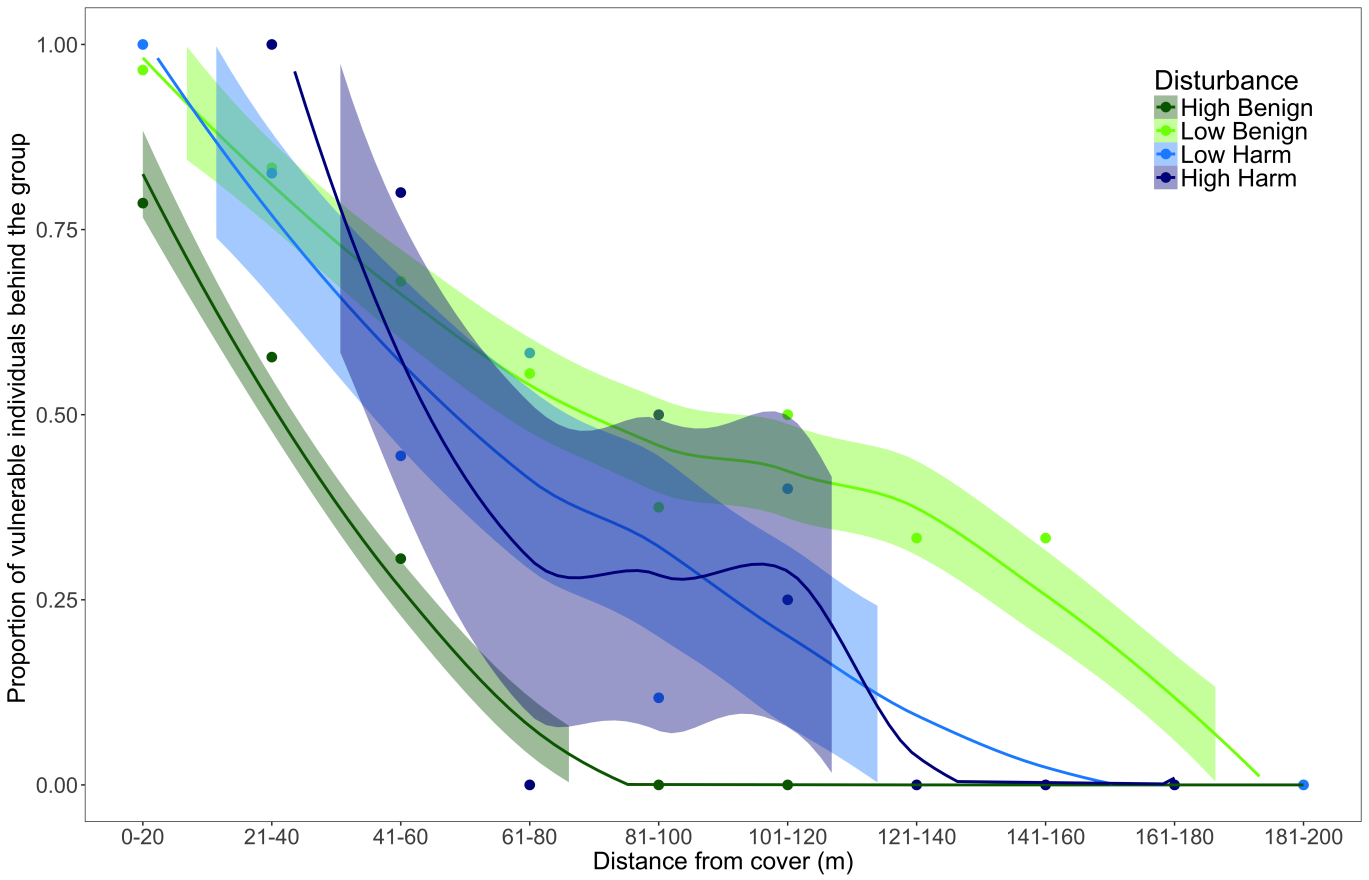
The relationship between the proportion of mothers, pouch young, and young-at-foot (vulnerable individuals) positioned closer to the forest edge and the group’s distance from cover. The relationships are plotted for each disturbance type with shaded regions reflecting confidence intervals (95%).

Distance from cover influenced the positioning of individuals in groups, with the majority of vulnerable individuals positioned closer to the forest edge when nearer to cover (Figure 4). The proportion of vulnerable individuals closer to the forest edge decreased as the group moved further from cover. This general trend was consistent across all disturbance types, however, significantly fewer vulnerable individuals were positioned closer to cover at *HB* than other for disturbance types, regardless of the group’s distance from cover (*LB: p* < 0.001, *LH: p* = 0.019, and *HH: p* = 0.018).

**Figure 5:**
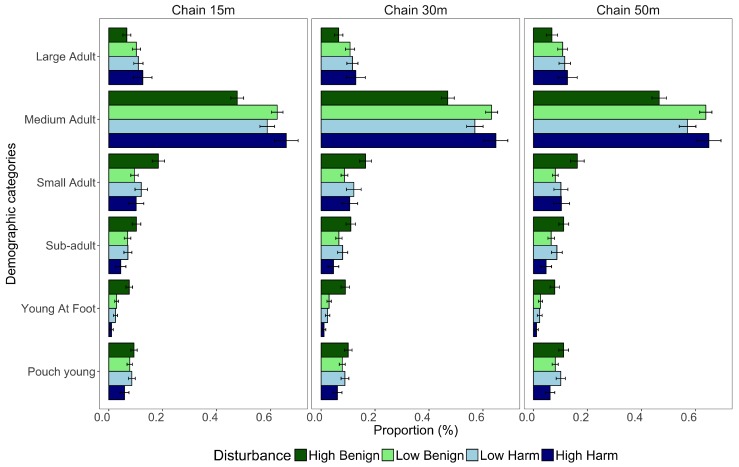
Demographic composition across different disturbance types using three measures of chain-rule to determine group membership; 15 m, 30 m, and 50 m. Demographic categories were large adult, medium adult, small adult, sub-adult, young-at-foot, and pouch young. Values are mean proportional contributions to groups, while error bars indicate standard errors.

Groups of kangaroos at *HB* had significantly larger portions of small adults than at *LB*, which was consistent for all chain-rules (15 m: *p* = 0.005, 30 m: *p* = 0.017, and 50m: *p* = 0.012) (Figure 5). The proportion of young-at-foot in each group was also significantly higher at *HB* than at all other disturbance types regardless of which chain-rule was implemented, 15 m (*LB: p* < 0.001, *LH: p* < 0.001, *HH: p* = 0.002), 30 m (*LB: p* < 0.001, *LH: p* < 0.001, *HH: p* = 0.002) and 50 m (*LB: p* = 0.001, *LH: p* = 0.002, *HH: p* = 0.001). Due to higher percentages of small adults and young at foot in groups at *HB* proportion of other demographic categories had to be reduced. This was evident at *HB* as medium adults contributed to a significantly lower proportion of the group than at all other disturbance types using the 15 m chain-rule (*LB: p* < 0.001, *LH: p* = 0.012, *HH: p* = 0.001). This trend was also observed using the 30 m and 50 m chain-rules, with significantly lower portions of medium adults at *HB* than at *LB* and *HH* (30 m: *p* < 0.001, *p* = 0.001; 50 m: *p* < 0.001, *p* = 0.001 respectively).

### 3.3. Landscape Responses

**Figure 6:**
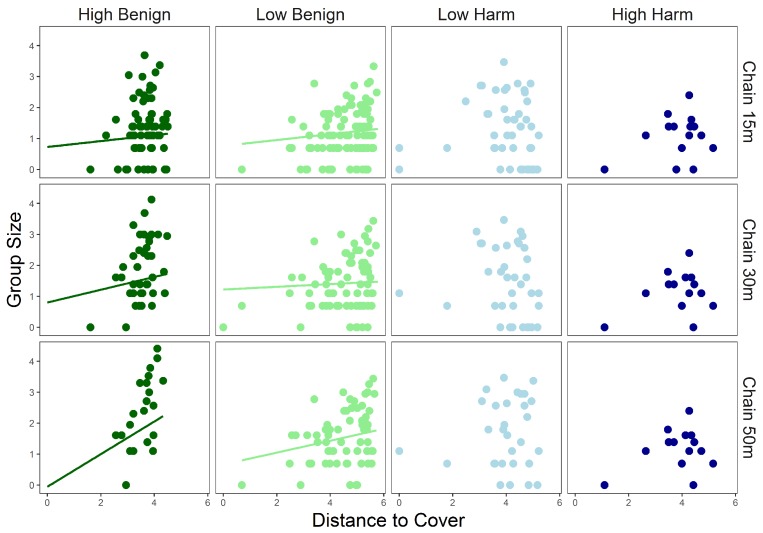
Relationship between logged distance to cover (m) on logged group size (n) as a function of human disturbance at each definition of chain rule (15 m, 30 m, and 50 m). Linear trend lines were plotted for significant relationships.

Distance to cover and group size was positively correlated at *HB* and *LB* sites, with the relationship strengthening as the chain-rule increased at *HB* sites (Figure 6, Table 2). No significant correlation between group size and distance from cover was detected at *LH* and *HH* sites (Figure 6, Table 2), which was consistent across all chain-rules.

We found that forage greenness varied significantly across disturbance types (*p* < 0.001) (Figure 3D). Mean greenness at *HB* was 35.76%; significantly greener than all other disturbance types (all comparisons, *p* < 0.001) and 1.23% higher than the next greenest disturbance type, *HH*, which had a mean of 34.53%. However, no correlation between forage greenness and group size was detected at any disturbance type. This was evident for all chain-rules (Table 3).

## 4. Discussion

We found that eastern grey kangaroos can respond behaviourally to both the frequency and intent of human disturbances. Although average group sizes varied little between human disturbance types, groups of larger sizes were consistently observed at benign sites relative to harmful sites, and were also influenced by frequency (higher with high benign but lower with high harm). Furthermore, these responses significantly altered the previously reported relationship between group size and distance from cover. At our study location, the typical antipredator response of forming larger groups when grazing further from the forest cover [35,36,37] broke down when human interactions with kangaroos were of harmful intent. Counter to our predictions, we detected no significant relationship between group size and distance to cover for kangaroos at low and high harm sites. However, the antipredator response of forming larger groups when grazing further from cover was detected when groups were subject to benign human interactions (both at low and high frequencies). Forming larger groups when grazing in open habitat has been hypothesised to assist with detecting and evading predators such as dingoes or foxes (according to the many-eyes hypothesis [38,39]), but leaves groups vulnerable to attack by human hunters whose success rate improves when clear site lines are obtained [35]. Hunters do not rely on an ambush attack and often go unnoticed by prey until the first shot is fired. Hunters are also able to fire shots in quick succession, allowing them to shoot several targets within the group, voiding the benefits of the dilution hypothesis [40].

One explanation of our findings is that kangaroos are modifying their antipredator behaviour in response to the novel threat posed by humans. Our results show that eastern grey kangaroos can maintain typical antipredator responses when humans are frequently present and their intent is benign, but that these responses are suppressed when humans act with aggression. Clearly kangaroos did not favour foraging in the open at harmful sites, as foraging densities were lower than at benign sites (either by choice or by being killed), but our findings suggest that the changes in responses were not driven by differences in density alone. Nor were they being driven by differences in resource quality as we did not detect any significant response to grass greenness. It has been well established that resource quality and quantity is an important factor in the selection of foraging habitat by eastern grey kangaroos [54,55,56], especially where grass is greener and therefore higher in energetic value [44,57,58]. Although resources at our high benign sites were significantly greener than at other sites, the difference was small (1.23%) and unlikely to greatly affect decision making at this magnitude. One recent study similarly reported no relationship between group size and distance to cover, possibly because resource availability was a positive driver of group size at their study site [44]. Although poisoning efforts targeting foxes and dingoes at our study location are frequent, low level predation effects (direct and indirect) are likely to be present.

Several prey species have been shown to exploit humans directly or indirectly to avoid predation or secure resources [69,70,71]. In line with our predictions, we found that eastern grey kangaroos within our study site were at higher densities in areas of frequent benign human activity. Although there may be unmeasured reasons why densities were higher at high benign sites, this finding implies that they can habituate to benign presence and may benefit from being shielded from persecution of harmful human activity [7]. In Australia, hunting native species without a permit is illegal, yet it is widely known that shooting is common in areas where encounters with other humans are rare, often on vacant land or private land where owners are not permanently living. However, in areas with high levels of human activity, hunting, both legally or illegally, is hazardous for recreationists and carries the risk of the shooter being reported to authorities. Additionally, it is possible that frequent human activity may suppress predation and provide sanctuary for kangaroos [48], although we have no direct evidence of this. With high levels of benign disturbance potentially deterring both natural predators and human hunters, it is difficult to disentangle their complementary effects. For example, we found that small adults and young-at-foot comprised a significantly greater proportion of groups at high benign sites than at harm sites. This suggests that raising of young may be easier at high benign sites, either through protection from shooting or predation. Juvenile mortality rates in eastern grey kangaroos can be high as they are subject to disease, malnutrition, exposure, and predation [72,73,74]. Where hunting is prevalent, juvenile mortality should increase due to increased stress and loss of parental care. Further research is required to track the causes of mortality across the disturbance types utilised in our study. This information is lacking with previous work on juvenile mortality was conducted at locations with benign human disturbance [75,76].

We found that eastern grey kangaroos modified their grouping behaviour and spatial dispersion in response to the intent and frequency of human disturbances at our study site. The plasticity of these responses alludes to cognitive learning in both forms; kangaroos habituating to the absence of consequence from a stimulus (human presence) at sites with benign human disturbance, and associating negative consequences from the same stimulus at neighbouring sites with harmful human disturbances. While our evidence for this is currently observational, further work will seek to clarify the causal effect of human presence on fear responses in these kangaroos. Behavioural plasticity may be instrumental to survival in rapidly changing environments, where human activities may offer both novel opportunities and significant risk. This study provides insight into how kangaroos are persisting in these complex landscapes and paves the way for long-term behavioural studies to investigate the mechanisms through which wildlife are persisting in landscapes shared with humans.

## Figures and Tables

**Table 1:** Results of statistical tests of skewness and kurtosis for the distribution of group size data from different disturbance types. Estimates and *p*-values are provided for each disturbance type expressed as abbreviation; HB (High Benign), LB (Low Benign), LH (Low Harm), and HH (High Harm) across chain-rules (15 m, 30 m, and 50 m).

Disturbance	Chain 15 m		Chain 30 m		Chain 50 m	
Type	*Skew*	*Kurtosis*	*Skew*	*Kurtosis*	*Skew*	*Kurtosis*
HB	3.72, *p* < 0.001	20.57, *p* < 0.001	3.59, *p* < 0.001	20.06, *p* < 0.001	2.63 *p* < 0.001	10.58, *p* < 0.001
LB	2.41, *p* < 0.001	10.64, *p* < 0.001	2.28, *p* < 0.001	9.42, *p* < 0.001	1.97, *p* < 0.001	7.25, *p* < 0.001
LH	2.72, *p* < 0.001	14.08, *p* < 0.001	1.76, *p* < 0.001	6.24, *p* = 0.001	1.35, *p* < 0.001	4.10, *p* = 0.046
HH	1.62, *p* < 0.001	6.01, *p* = 0.004	1.42, *p* < 0.001	4.91, *p* = 0.021	2.27, *p* < 0.001	9.83, *p* < 0.001

**Table 2:** Results of GLMMs testing for effect of disturbance (High Benign, Low Benign, High Harm, High Harm) and logged distance to cover on log-transformed group size. Each model considers the data using a different chain-rule (15 m, 30 m, or 50 m) using negative binomial errors. Effects (β) are presented with 95% confidence intervals (method: Wald) which are highlighted in bold when intervals do not cover zero. Categorical fixed effects are relative to reference level (High Benign).

Fixed Effects	Chain 15 m	Chain 30 m	Chain 50 m
	β *(CI)*	β *(CI)*	β *(CI)*
Intercept (HB)	−0.88 (−2.02, 0.25)	−1.19 (−2.40, 0.01)	**−1.64 (−3.10, −0.18)**
LB	−0.04 (−1.58, 1.50)	0.72 (−0.74, 2.17)	1.00 (−0.64, 2.65)
LH	1.19 (−0.21, 2.58)	**1.97 (0.48, 3.46)**	**1.88 (0.01, 3.75)**
HH	0.11 (−2.68, 2.91)	0.60 (−1.97, 3.17)	1.05 (−1.48, 3.59)
HB:Cover	**0.31 (0.01, 0.61)**	**0.50 (0.17, 0.83)**	**0.69 (0.29, 1.08)**
LB:Cover	**0.23 (0.01, 0.46)**	**0.18 (0.02, 0.38)**	**0.24 (0.02, 0.46)**
LH:Cover	−0.01 (−0.23, 0.21)	−0.10 (−0.33, 0.14)	0.07 (−0.22, 0.35)
HH:Cover	0.21 (−0.41, 0.83)	0.19 (−0.37, 0.75)	0.19 (−0.34, 0.72)
**Random effects**	**σ (obs)**	**σ (obs)**	**σ (obs)**
Session	1.53 × 10${}^{-12}$ (234)	5.71 × 10${}^{-12}$ (166)	1.83 × 10${}^{-12}$ (130)

**Table 3:** Results of LMMs testing for effect of disturbance (High Benign, Low Benign, Low Harm, High Harm) and forage greenness on group size. Each model considered the data using a different chain rule (15 m, 30 m, or 50 m). Effects (β) are presented with 95% confidence intervals (method: Wald) which are highlighted a bold when intervals do not cover zero. Categorical fixed effects are relative to reference level (High Benign).

Fixed Effects	Chain 15 m	Chain 30 m	Chain 50 m
	β *(CI)*	β *(CI)*	β *(CI)*
Intercept (HB)	0.72 (−2.48, 3.93)	−2.06 (−6.93, 2.81)	−2.20 (−8.30, 3.90)
LB	0.00 (−5.31, 5.31)	3.21 (−4.06, 1.48)	2.18 (−6.63, 10.99)
LH	−3.29 (−8.38, 1.79)	−0.39 (−7.18, 6.39)	−5.54 (−14.23, 3.14)
HH	6.43 (−5.57, 18.44)	12.60 (−1.35, 26.55)	12.68 (−1.63, 26.98)
HB:Green	−0.02 (−0.07, 0.11)	0.11 (−0.03, 0.25)	0.13 (−0.04, 0.30)
LB:Green	−0.01 (−0.11, 0.14)	0.01 (−0.15, 0.16)	0.05 (−0.14, 0.23)
LH:Green	0.11 (−0.01, 0.22)	0.11 (−0.02, 0.25)	0.27 (−0.01, 0.45)
HH:Green	−0.17 (−0.51, 0.16)	−0.27 (−0.64, 0.11)	−0.27 (−0.64, 0.11)
**Random effects**	**σ (obs)**	**σ (obs)**	**σ (obs)**
Session	0.00 (234)	1.73x10${}^{-14}$ (166)	0.131 (130)
